# Infected Non-Union of the Distal Femur

**DOI:** 10.7759/cureus.12613

**Published:** 2021-01-11

**Authors:** Kishore Vellingiri, Nagakumar J S

**Affiliations:** 1 Orthopaedics, Sri Devaraj Urs Academy of Higher Education and Research, Kolar, IND

**Keywords:** nonunion of distal femur

## Abstract

The reported levels of non-union in the lateral locking plate differ widely, with some early studies showing rates of less than 6% and up to 17%-21% in more recent studies. We report a case where better results were shown by a non-union treated with distal femoral nailing with allogenic grafting.

## Introduction

Three to six percent of adult femoral fractures and 0.4% of all fractures are distal femoral fractures, with a bi-modal distribution usually consisting of younger patients with high-energy injuries and older patients with minor trauma fractures [1]. The reported levels of non-union in the lateral locking plate differ widely, with some early studies showing rates of less than 6% and up to 17%-21% in more recent studies [2-4]. We report a case where better results were shown by a non-union treated with distal femoral nailing with allogenic grafting.

This case study was presented as poster presentation at the 39th Annual Conference of Kerala Orthopaedic Association Conference (KOACON 2020) held on 24th to 26th January 2020 in Kerala, India. The abstract of this article is published in the online supplement of the conference journal.

## Case presentation

**Figure 1:**
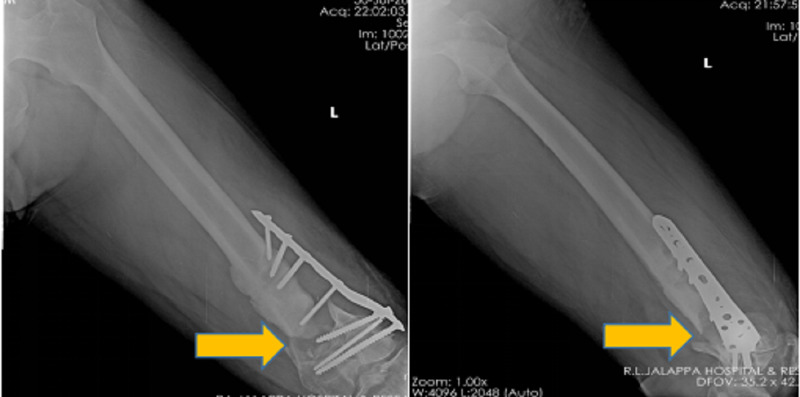
Plain antero-posterior and lateral left thigh radiograph, indicating non-union of left distal femur with locking compression plate in situ.

**Figure 2:**
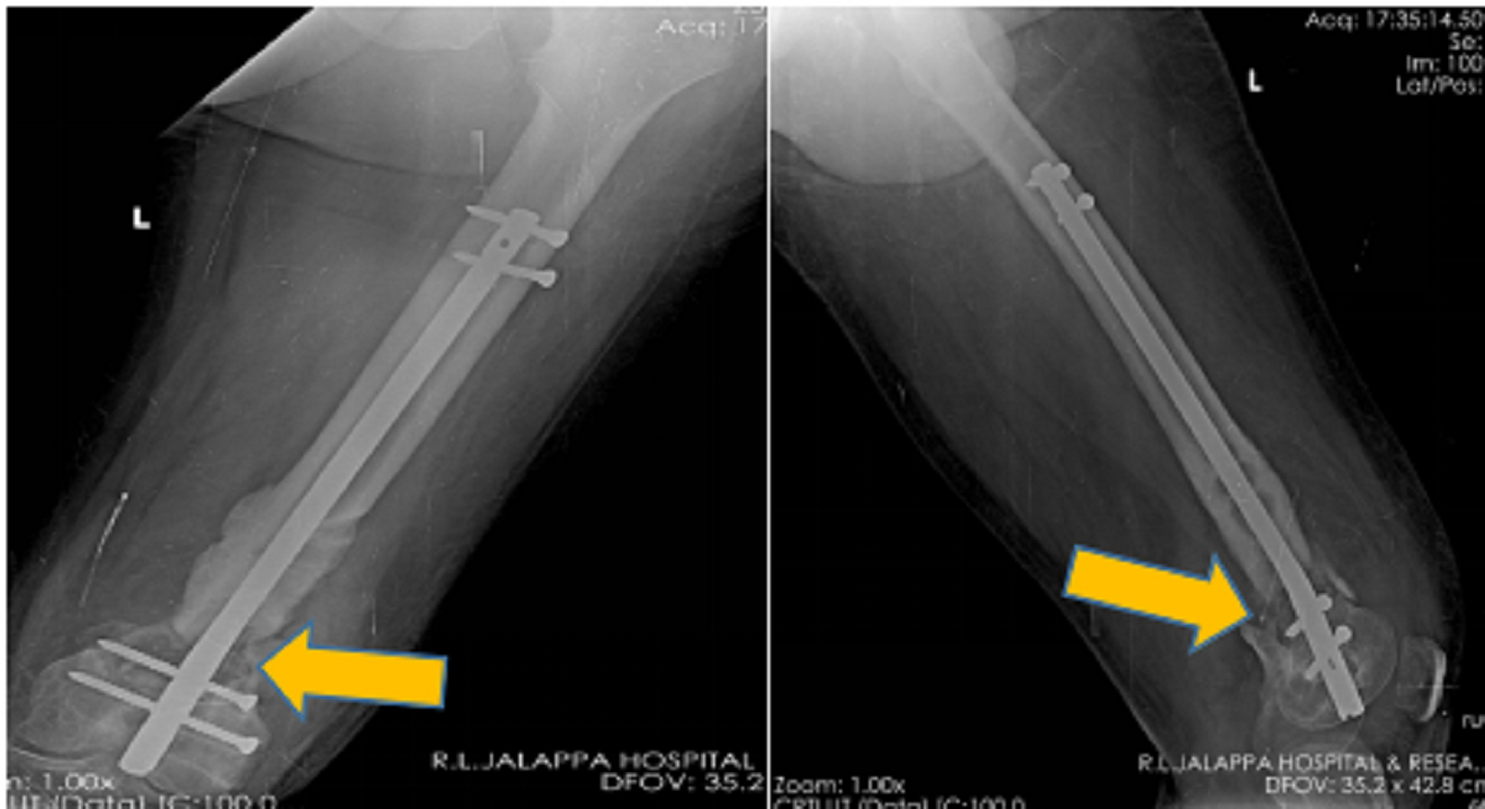
Post-operative left thigh antero-posterior and lateral view radiograph showing removal of the locking compression plate with distal femoral nail fixation with allogenic bone grafting.

**Figure 3:**
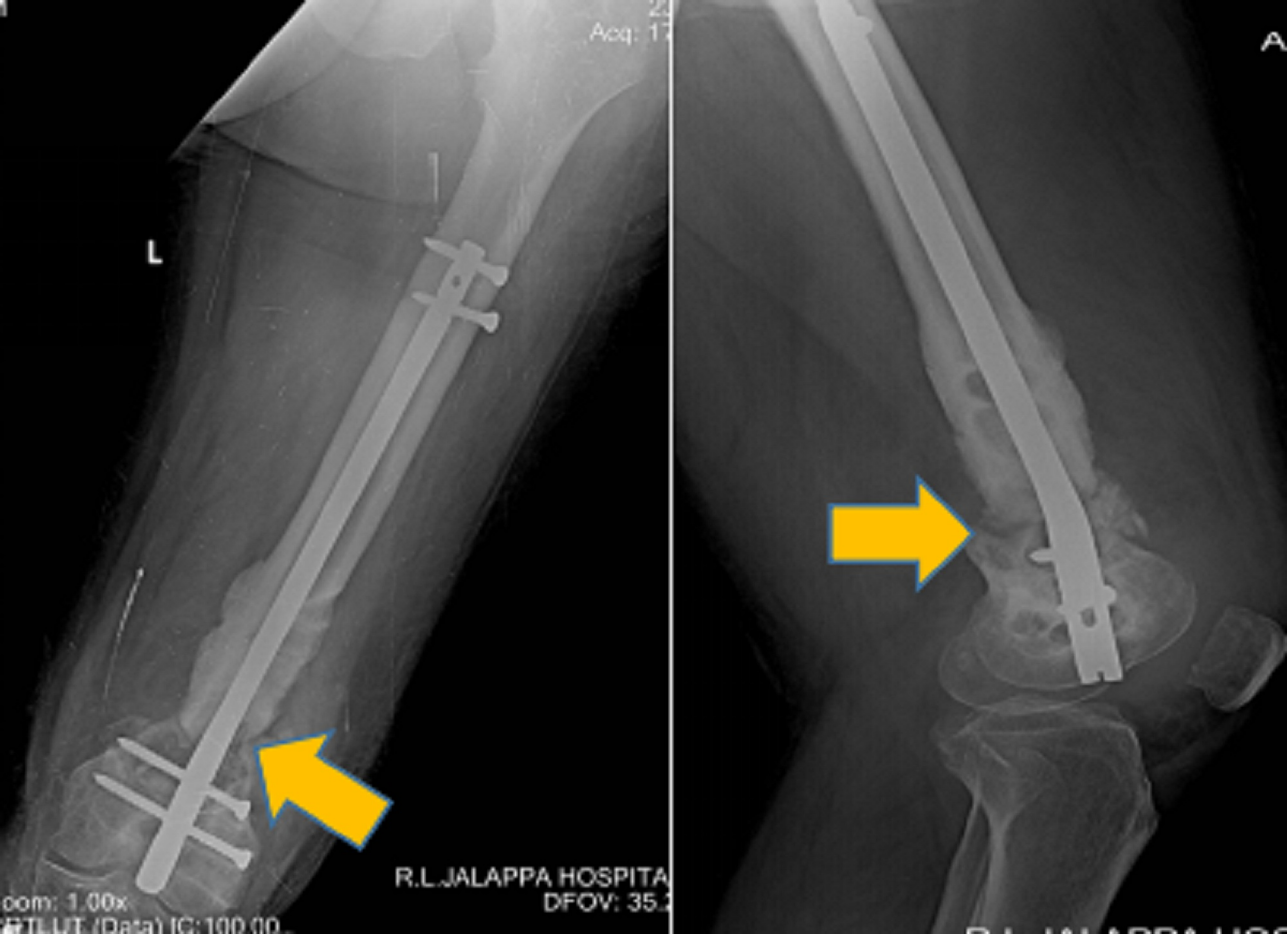
Six-month post-operative, plain antero-posterior left thigh radiograph and lateral view showing fracture uniting with distal femoral nail in place

We present a 45-year-old male to our tertiary care facility in Kolar, India. Three years old infected non-union of distal femur with locking compression plate in place treated in various hospitals. With the support of the stick, the patient was partly weight-bearing. When examined, the distal left thigh portion - swelling, tenderness and abnormal mobility was present. The range of motion was 45-120 degrees of flexion at the left knee joint. The extensor lag was 45 degrees. There were no distal neurovascular deficits found. The patient was sent for a left thigh radiograph showing non-union distal femur with locking compression plate in in-situ as shown in Figure 1. The patient underwent locking compression plate removal with distal femoral nail fixation with allogenic bone grafting after written/informed consent. Patient was put on six weeks above knee slab immobilization. Hospital treatment: the patient received cephalosporin group intravenous antibiotics twice daily for seven days, followed by oral cephalosporin antibiotics twice daily for seven days, depending on the patient’s post-operative weight. In Figure 2, post-operative radiography of the left thigh was seen. There was an uneventful post-operative time. The site of surgery healed well. The range of motion at the knee joint was 0-120 degree flexion at discharge. After six weeks, partial weight-bearing was started. At the last follow-up, six months radiography, the fracture site is uniting as shown in Figure 3 and the patient is partially weight-bearing without any trouble.

## Discussion

The two main components of the fracture union are discussed in the management of every non-union: structural support and biology [5]. The risk factors for distal femoral non-union concluded that the use of a longer plate reduces the probability of non-union. The extend of the injury and related co-morbid conditions such as diabetes mellitus and obesity are the deciding factors for non-union [6]. The prognostic risk factors for non-union in distal femoral fractures treated with lateral locking plate are obesity, open fracture, the prevalence of infection and the use of stainless steel, irrespective of the various patterns in how surgeons intervene in non-union management [7]. The combination of plate configuration and material variable has a highly important impact on the probability of non-union independently of any other build variable in the treatment of distal femur fractures with lateral locking plates [8]. Open fracture, diabetes mellitus, smoking, increased body mass index and shorter plate length were the established risk factors for re-operation to facilitate union and complications. Most variables are beyond the influence of the surgeon, but are useful when contemplating prognosis. A technological consideration that can reduce the probability of fixation failure is the use of relatively long plates [6]. Combining a locking plate fixation with the bone grafting technique by using an allograft strut to sustain the defect of the metaphyseal medial bone and auto-grafts provides a strong union and a good functional results by improving biology and providing good structural support to distal femur in the management of resistant non-union of the distal femur [9]. Further enhanced reduction, correction of the medial bone defect and biological help with bone grafting are the key principles for resolving a distal femoral non-union. The combination of a medial strut graft or a medial column plate may be very useful in these non-unions on the mechanical side [10]. For the treatment of infraisthmal femoral shaft non-union, poller screw and additional interlocking screws, along with intramedullary nailing exchange, can be an efficient and secure alternative [11]. For the treatment of atrophic distal femur non-union with bone defect, J shaped iliac crest bone graft combined with double plate resulted in less maximal tension and less displacement than a J shaped iliac crest bone combined with lateral locking plate or double plate only graft. Less surgical trauma, early rehabilitation exercise after surgery, a high rate of bone healing and a satisfactory rate of functional recovery were associated with this procedure [12]. An attractive alternative seems to be the idea of polytherapy for the treatment of non-union, namely the simultaneous application of the three fundamental elements of the diamond concept, osteoprogenitor cells, growth factor and osteoconductive scaffold; however, it is desirable to verify this technique with further research [13]. By stimulation of fracture healing, early cases of delayed/non-union with adequate mechanical stability and biologically active bone can be controlled. Usually, late presenting non-union involves revision of the construct of fixation and stimulation of the callus to trigger fracture union [14]. The distal femoral nail and a less invasive stabilization device were good in terms of technique and outcome for the treatment of distal femoral fractures. They were also superior in terms of infection and axial malalignments to the condylar plate. The two minimally invasive implants were good in terms of technique and outcome for the treatment of distal femoral fractures and did not vary substantially in epidemiology, fracture type, conversion procedures, infection rate, malalignments and subjective and objective results at one year follow up [15]. For infected non-union distal femur, the patient in our study had a successful clinical and functional outcome following allograft with distal femoral nailing.

## Conclusions

Compared to the load-bearing system in distal femur non-union, we illustrate the significance of load-sharing devices with better practical performance. For both the patient and the treating surgeon, rebuilding bone defects is a long, demanding operation. Bone allograft is particularly unusual for contaminated distal femoral no-union, given the possible complications and their potential effects on the patient’s functional status when informing patients about this operation.
